# Blind spectral unmixing for characterization of plaque composition based on multispectral photoacoustic imaging

**DOI:** 10.1038/s41598-023-31343-y

**Published:** 2023-03-13

**Authors:** Camilo Cano, Catarina Matos, Amir Gholampour, Marc van Sambeek, Richard Lopata, Min Wu

**Affiliations:** 1 Department of Biomedical Engineering, Eindhoven University of Technology, De Rondom 70, Eindhoven, the Netherlands; 2 Department of Surgery, Catharina Ziekenhuis Eindhoven, Michelangelolaan 2, Eindhoven, the Netherlands

**Keywords:** Medical imaging, Biomedical engineering

## Abstract

To improve the assessment of carotid plaque vulnerability, a comprehensive characterization of their composition is paramount. Multispectral photoacoustic imaging (MSPAI) can provide plaque composition based on their absorption spectra. However, although various spectral unmixing methods have been developed to characterize different tissue constituents, plaque analysis remains a challenge since its composition is highly complex and diverse. In this study, we employed an adapted piecewise convex multiple-model endmember detection method to identify carotid plaque constituents. Additionally, we explore the selection of the imaging wavelengths in linear models by conditioning the coefficient matrix and its synergy with our unmixing approach. We verified our method using plaque mimicking phantoms and performed ex-vivo MSPAI on carotid endarterectomy samples in a spectral range from 500 to 1300 nm to identify the main spectral features of plaque materials for vulnerability assessment. After imaging, the samples were processed for histological analysis to validate the photoacoustic decomposition. Results show that our approach can perform spectral unmixing and classification of highly heterogeneous biological samples without requiring an extensive fluence correction, enabling the identification of relevant components to assess plaque vulnerability.

## Introduction

Atherosclerosis is a systemic disease characterized by the accumulation of highly modified heterogeneous biomaterials inside the artery walls, forming plaque lesions that narrow the luminal size^1–3^. The rupture of vulnerable carotid plaques is considered one of the main causes of ischemic cerebrovascular disease and a leading source of mortality and morbidity worldwide^2, 4–6^. Previous studies have explored the formation and evolution of plaques and how they acquire the connotation of ‘vulnerable’ or ‘prone to rupture’^5, 7–10^. It has been found that the development of lipid-rich regions inside the plaque triggers an immune response from the body, resulting in tissue degradation and the formation of a thin fibrous cap that separates the lumen of the plaque material^6, 10, 11^. Accordingly, the pathological assessment of vulnerable plaque relies on the identification of lipid-rich necrotic areas and the thickness of the thin fibrous cap^2, 6^. Further studies have shown that intra-plaque hemorrhage is another relevant indicator of vulnerability in plaques^12–14^. The presence of intra-plaque hemorrhage is associated with either blood infiltration through the fibrous cap or the formation of new micro-vascularization^13^, both equally concerning. In the case of lumen infiltration, there is a high possibility of rupture or material infiltration that may lead to clogging formation^15^. On the other hand, neo-vascularization implies inflammation and the delivery of more macrophages and other biological constituents that promote plaque progression^13^.

One of the principal treatments for ischemic cerebrovascular events is carotid endarterectomy surgery, in which the plaque is removed from the carotid to prevent its rupture and clot formation^11, 16^. However, several studies indicate that a large number of patients that undergo surgery didn’t actually have a vulnerable plaque, and treatment through medication would have been possible^2, 11, 17^. Overtreatment is partially due to the current diagnosis based on the grade of stenosis, a geometrical relationship between the transversal size of the lumen and the size of the carotid^18^. As an alternative, some studies illustrate the benefits of using plaque composition as an additional criterion for plaque vulnerability assessment^2, 10, 11, 15^. Plaque composition provides valuable information on the possible mechanical characteristics of the plaques and their progression; specifically, lipid pools and hemorrhage promote macrophage infiltration, causing plaque growth and plaque instability^15^.

Ultrasound (US) and Computed Tomography (CT) imaging are the core imaging techniques used in the medical diagnosis of vulnerable plaques. Although they provide an accurate estimation of the plaque geometry, the usual approach does not provide information on the plaque composition. Some studies exemplify the use of magnetic resonance imaging (MRI)^12, 19^, phase-contrast computed tomography^20, 21^, and optical-based spectroscopy techniques^22, 23^ to determine the composition of carotid plaque. However, these techniques have disadvantages like the use of ionizing radiation, the impossibility of use in patients with implants, and limited penetration, respectively^11^. As an alternative, another promising image technique that provides composition information is multispectral photoacoustic imaging (MSPAI)^24–26^. In MSPAI, pulse light is used to illuminate the tissue, which will selectively absorb part of the optical energy that leads to the generation of an acoustic pressure wave due to thermoelastic expansion, detectable with conventional US probes^27^. The most outstanding feature of MSPAI is its optical contrast, allowing material determination from the characteristic absorption spectra of tissue while reaching imaging depths up to several centimeters, which clearly surpasses that of most purely optic techniques. Nonetheless, the accurate analysis of PA spectral modulation in patients remains a challenge due to tissue heterogeneity and inter-subject variability^28, 29^.

Photoacoustic (PA) signals are proportional to the product of the local fluence and the absorption coefficient of the tissue of interest^30^. To identify imaged components, MSPAI relies on the response of tissue chromophores, the optical absorbing molecules, to different wavelengths. However, the modulation of the PA signals in volumetric samples does not match with literature absorption coefficients due to the effect of local fluence variations, also called spectral coloring. It has been demonstrated that without an accurate correction of light fluence, linear unmixing approaches are not feasible for complex heterogeneous samples, usually leading to erroneous estimations^31^. Besides, the exact composition of the plaque and surrounding tissue is unknown, increasing the complexity of fluence estimation; thus, we need new methods that can work without prior knowledge of the absorption spectra of the sample. The most common solution is the use of blind unmixing techniques, where linear models are employed to fit the acquired data and estimate the constituent spectra (called endmember) and their proportion distribution^32, 33^. The endmembers correspond to the representative PA spectra of the different absorbers in the sample, and the proportion maps determine the percentage of each endmember per pixel. Considering the high heterogeneity of biological tissue, the problem at hand requires an unmixing method that does not work under the assumption of pure pixels consisting of a single component. In this work, we adapted a Piecewise Convex Multiple-Model Endmember Detection (PCOMMEND)^34^ for the unmixing of MSPAI that deals with the nonhomogeneous illumination conditions of photoacoustic imaging (PAI). PCOMMEND is a pixel-based method without pure pixel assumption that extends the linear mixing model to multiple sets of endmembers, allowing the solution of non-convex problems. This property supports the unmixing of datasets with limited bandwidth and fluence variations, problems that greatly hinder the use of linear unmixing techniques. By splitting the data into multiple regions with similar fluence based on the amplitude of the signal, it is possible to obtain a better fitting of the endmembers in the model and find an average representation of the spectra in the sample. In contrast to other promising blind unmixing approaches like non-negative matrix factorization (NNMF), the multiple-model approach obtains a non-convex solution by optimizing individual convex problems, making the solution more stable^34^.

One of the main challenges for the spectral analysis of multiple materials in biological samples is that spectral markers can be distributed over a wide spectral range, making in-vivo implementation impractical due to the low repetition rate of tunable lasers. Acquisition time optimization is essential for MSPAI to be viable in medical applications; hence, it is necessary to optimize the number of imaging wavelengths to identify as many components as possible. Different approaches for wavelength selection have been proposed, with some focusing on optimizing in-vivo imaging conditions^35, 36^, while others optimize the model fitting^37–40^. In this work, we aim to select the most suitable set of wavelengths for an accurate unmixing of the data; therefore, we introduce an additional technique designed to minimize the number of variables required for solving linear models. For this purpose, one of the most widely used methods for imaging wavelength selection is model conditioning, which can be achieved by optimizing the model’s coefficient matrix^38, 39^.

In this study, we demonstrate unmixing of multi-wavelength PA signals of carotid plaques based on a multiple-model approach (PCOMMEND), demonstrating the first implementation of such an approach in the unmixing of spectral signals from highly heterogeneous biological tissue. The method performance is first evaluated quantitatively on a plaque mimicking phantom. Next, MSPAI is performed on ex-vivo carotid endarterectomy samples, and the unmixing results are validated by comparison with histology. Moreover, we optimize (and limit) the number of imaging wavelengths through the conditioning of the model to investigate how our approach can work for in-vivo scenarios. Our results show that it is possible to identify multiple plaque constituents with a significant correlation with histology staining. Besides, the capability of splitting the initial dataset into multiple models has proven to support the blind unmixing of low amplitude signals. Four plaque materials were classified as repeatable over the different samples. Two of them, hemorrhages and lipids, have a significant diagnostic value. The other two, collagen and smooth muscle cells (MSC), may become relevant in light of recent studies^41–43^.

## Experiment description

**Figure 1:**
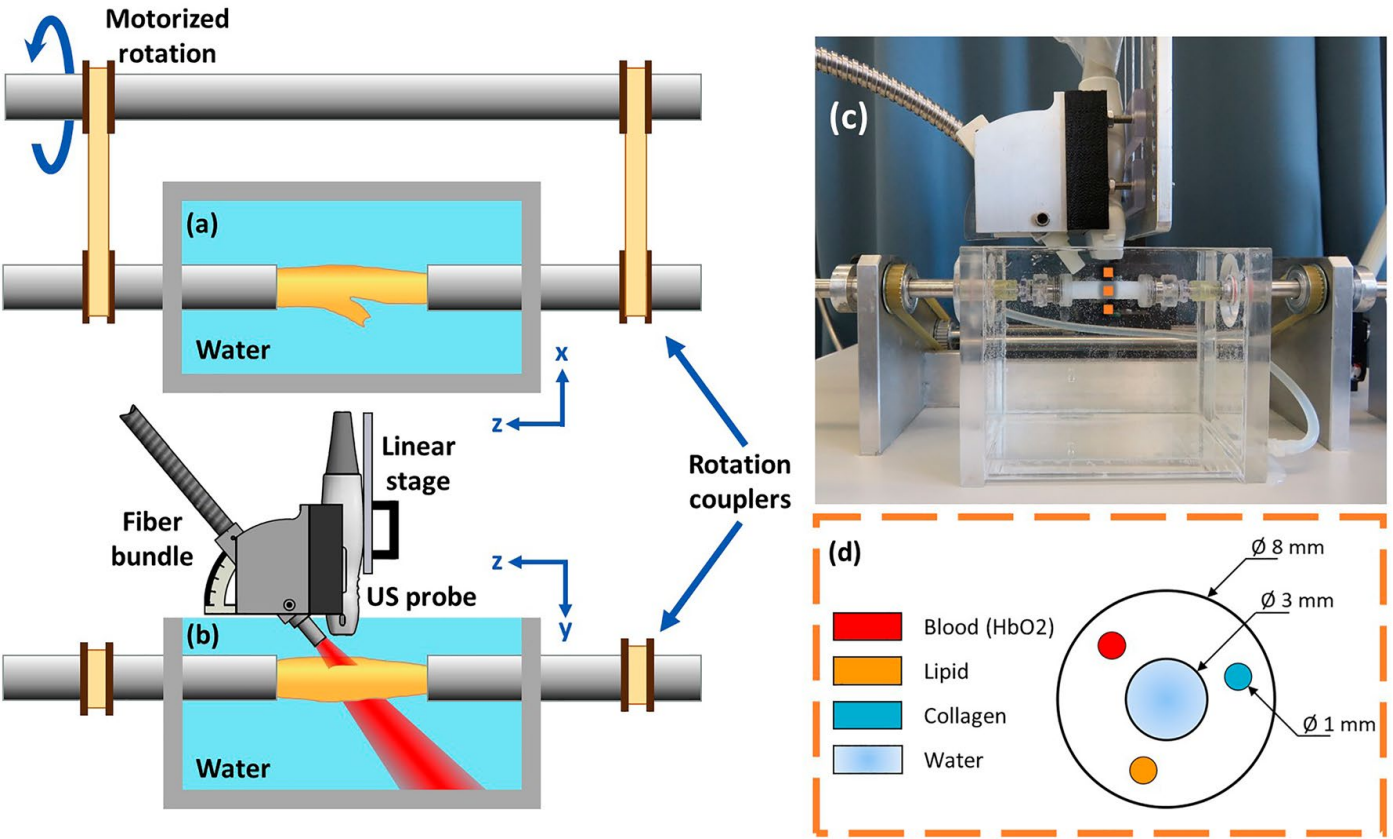
Imaging setup. (**a**) Schematic of the imaging setup top view and (**b**) side view. The fiber bundle and probe are attached to a linear movement stage for positioning, and the sample can be rotated for multiple perspective acquisition. (**c**) Photograph of the photoacoustic setup side view with a PVA vessel phantom. (**d**) Schematic of the three-channel phantom used for the method validation.

The imaging setup is presented in Fig. 1. Light radiation was generated with a tunable pulsed optical parametric oscillator laser (OPOTEK radiant HE 355 LD, Carlsbad-California, USA) and delivered using a custom-made bundle fiber (CeramOptec, Bonn, Germany). Acoustic pressure was measured using a Verasonics Vantage system with a linear array probe (L11-5, Kirkland-Washington, USA). A vessel mimicking polyvinyl alcohol (PVA) phantom with three inclusions was designed to validate the unmixing and wavelength selection techniques (Fig. 1d). The three channels in the phantom were filled with oleate cholesterol, porcine blood, and tissue obtained from an endarterectomy surgery to mimic the plaque lesions. The same setup was used to scan ex-vivo carotid endarterectomy plaques obtained from the Catharina hospital in Eindhoven. During the measurement, phantoms and endarterectomy plaques were immersed in water and mounted at the in- and outlet, allowing rotation of the sample. Phantom MSPAI was performed at 162 wavelengths (from 500 to 1300 nm in steps of 5 nm) with an average of 20 frames per PA acquisition and from 5 different perspectives between 0° and 120° (in steps of 30°), emulating the achievable imaging field of view of a patient. Complementary ultrasound images were acquired using plane-wave acquisition with 21 steering angles for geometrical comparison. We used delay-and-sum beamforming for image reconstruction and average amplitude estimation from different perspectives to increase the contrast-to-noise ratio (CNR), signal-to-noise ratio (SNR), and field of view.

## Results

### Feasibility study on phantom data

**Figure 2:**
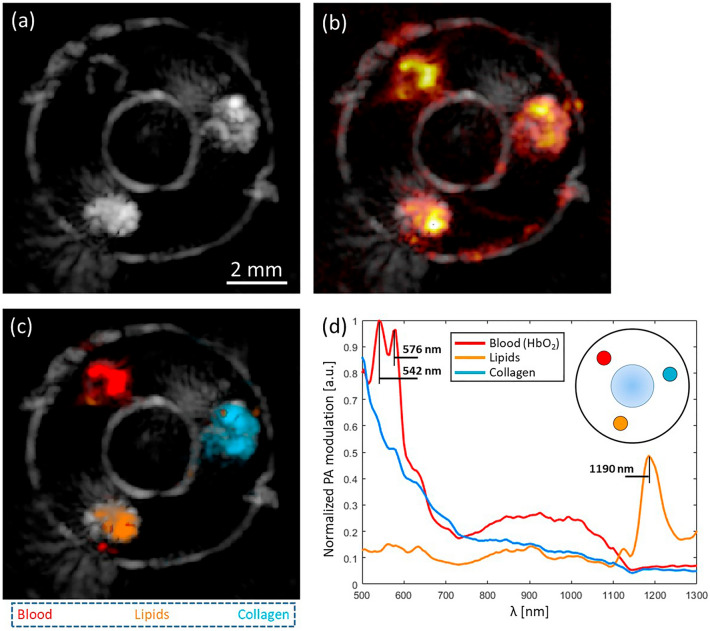
Three-channel PVA vessel mimicking phantom. (**a**) US image, (**b**) PA-US overlay image acquired at 550 nm. (**c**) Segmented regions obtained from the proportion maps of blood, which is mainly constituted of oxygenated hemoglobin (HbO_2_), lipids, and collagen using 162 imaging wavelengths. (**d**) Endmembers calculated from the PA signal of each material (with water absorption correction). Abundance map coincides with the phantom geometry and the endmembers confirm the composition of the channels.

The US and PA images of the phantom are shown in Fig. 2a,b. The dataset of 162 wavelengths was processed using the unmixing method proposed to determine the endmembers and proportion maps from the signal. Figure 2d shows the endmembers of the three channels where the main features of oxygenated hemoglobin (peaks at ≈ 542 nm and ≈ 576 nm) and cholesterol (peak at ≈ 1190 nm) are recognizable, and the remaining one fits into the spectrum of collagen^44, 45^. Additional endmembers were found for the background and PVA, but since they do not provide additional biological information and for the sake of clarity, they are not displayed. In Fig. 2c, the proportion map for the endmembers in Fig. 2d illustrates the potential of the unmixing method to differentiate the material distribution of biological samples.

The mean percentage error of the regions misidentified after unmixing is presented in Table 1 (first column). It corresponds to the percentage error of the area between each material prediction with the ground truth defined by the morphology of the channel obtained with US. Overall, the error is below 10%, indicating a proper determination of the material distribution. The higher error of 7.6% for lipids is most likely due to spectral coloring artifacts that have a higher effect on low amplitude signals. The errors for the blood and collagen predictions are minor and mainly due to reconstruction artifacts associated with the limited aperture of the transducer.

**Table 1:** Error estimation in phantom measurements.

Material	Mean percent error in the determination of the components’ location (± SD) [%]	Mean percent error between 162 and 6 wavelengths imaging (± SD) [%]
Blood	3.2 ± 1.1	6.2 ± 6.5
Lipid	7.6 ± 2.3	3.5 ± 6.2
Collagen	1.7 ± 1.2	10 ± 24

First column is the mean percent error for each channel’s area between the MSPAI prediction and the channel’s area calculated with US. The second column is the mean percent error in proportion determination between 162 and 6 wavelengths inside the channels area.

### Wavelength selection

**Figure 3:**
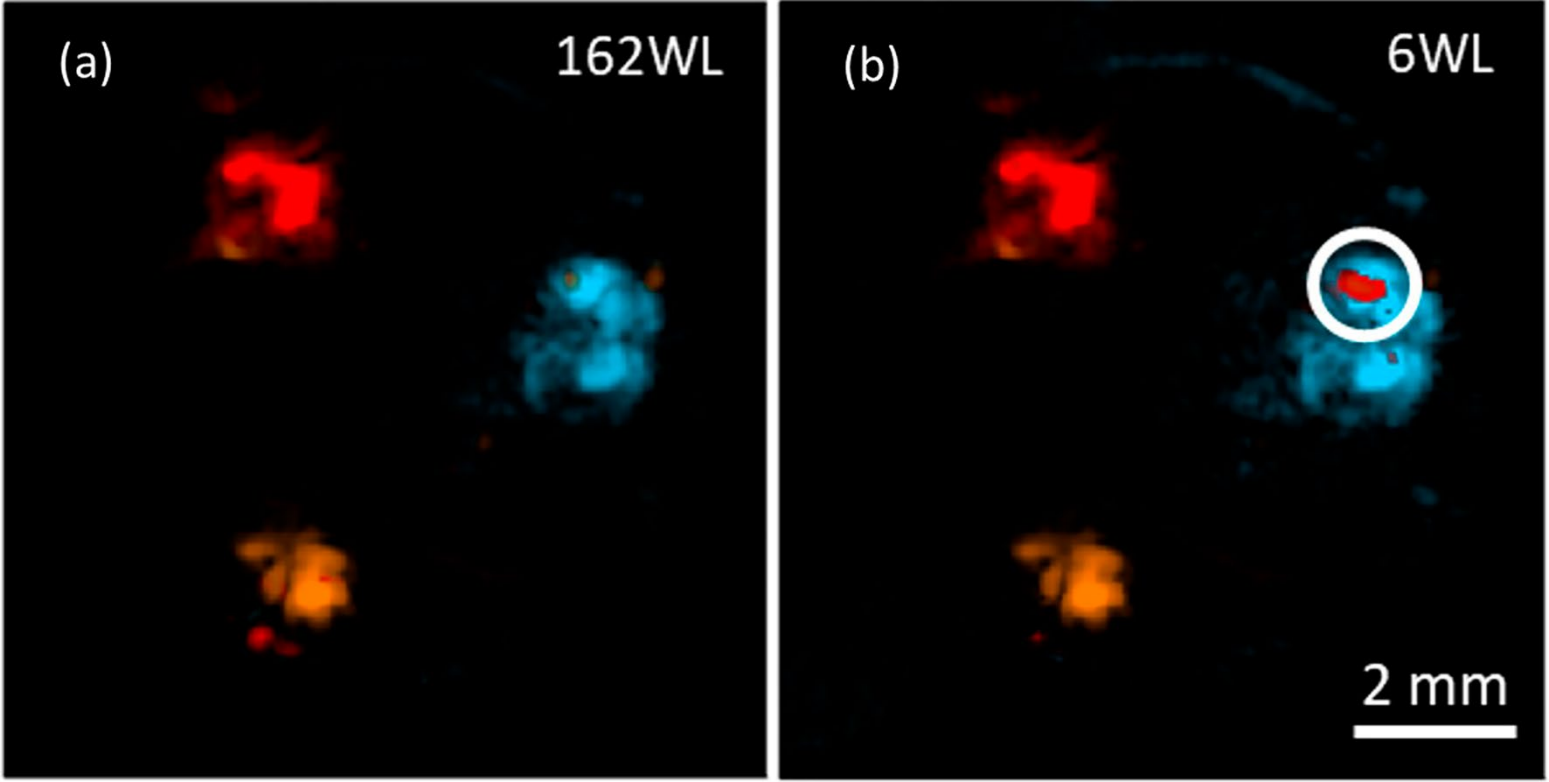
Wavelength selection analysis. (**a**) is the proportion map obtained using 162 wavelengths and (**b**) the result using an optimized set of 6 wavelengths.

Considering that the same approach can be used in specific spectral windows, we evaluate the performance of our wavelength selection in the phantom data out of a broad spectral range from 500 to 1300 nm, including as many spectral markers as possible. Given that the maximum number of endmembers required to unmix the phantom data was six, the data was optimized for the same number of wavelengths. In Fig. 3a,b, we show the unmixing results using 162 and 6 wavelengths, respectively, where the composition and distribution prediction for the three channels corresponds between both datasets. The selected set of wavelengths for the phantom data is: [500 nm, 560 nm, 680 nm, 780 nm, 940 nm, 1020 nm].

Since the blind unmixing problem is solved using a least square minimization, unmixing with a limited number of wavelengths is more sensitive to outliers and noise. Table 1 (second column) summarizes the mean error between the proportions obtained with 162 and 6 wavelengths within the area of the channels. Proportion estimations take a value between 0 and 1, and the mean error for the three channels is below 10%. It indicates good reproducibility in the unmixing even when the number of wavelengths decreases. The standard deviation is below 10%, except for the plaque collagen channel, where blood is erroneously detected.

### Endarterectomy plaques data

We present three selected cases of plaque morphologies that exemplify the diagnostic relevance of MSPAI: One stable plaque with low lipid content, and two vulnerable plaques with a lipid-rich necrotic core in one sample and an intraplaque hemorrhage in the other.

**Figure 4:**
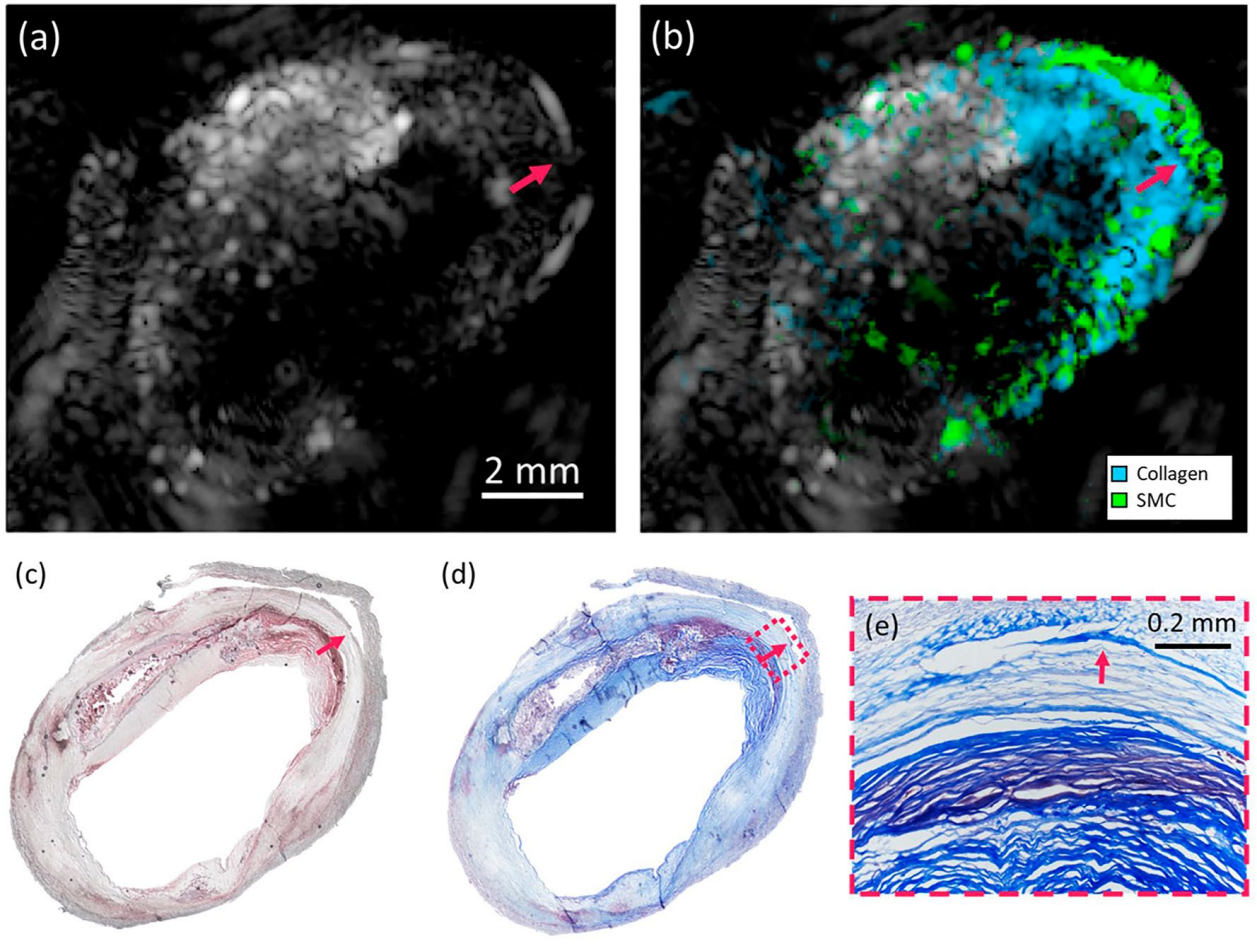
Endarterectomy plaque with low lipids concentration. (**a**) is the US image and (**b**) is the proportion map for collagen and SMC using selected wavelengths. (**c**) Shows the ORO staining of the sample, indicating low lipid content. (**d**) MT staining, there is an interface that indicates the presence of collagen in the imaged region. (**e**) MT stain (magnification × 100) shows a decrease in collagen content near the material interface, which is effectively detected in the proportion map (**c**).

Figure 4 shows one sample where the imaging region consists mainly of collagen and SMC. A red arrow indicates the location of the interface between the collagen and collagen-free regions, which is clearly distinguishable in the unmixing map (Fig. 4b) in contrast with the US image (Fig. 4a), where no features can be observed. Oil Red O (ORO) histology in Fig. 4c confirms that there is no lipid, and Masson Trichrome (MT) shows the location of the collagen fibers in Fig. 4d. A 100X magnification of the collagen interface is shown in Fig. 4e, confirming that despite the decrease in collagen density, MSPAI unmixing accurately determines the location of the different materials.

**Figure 5:**
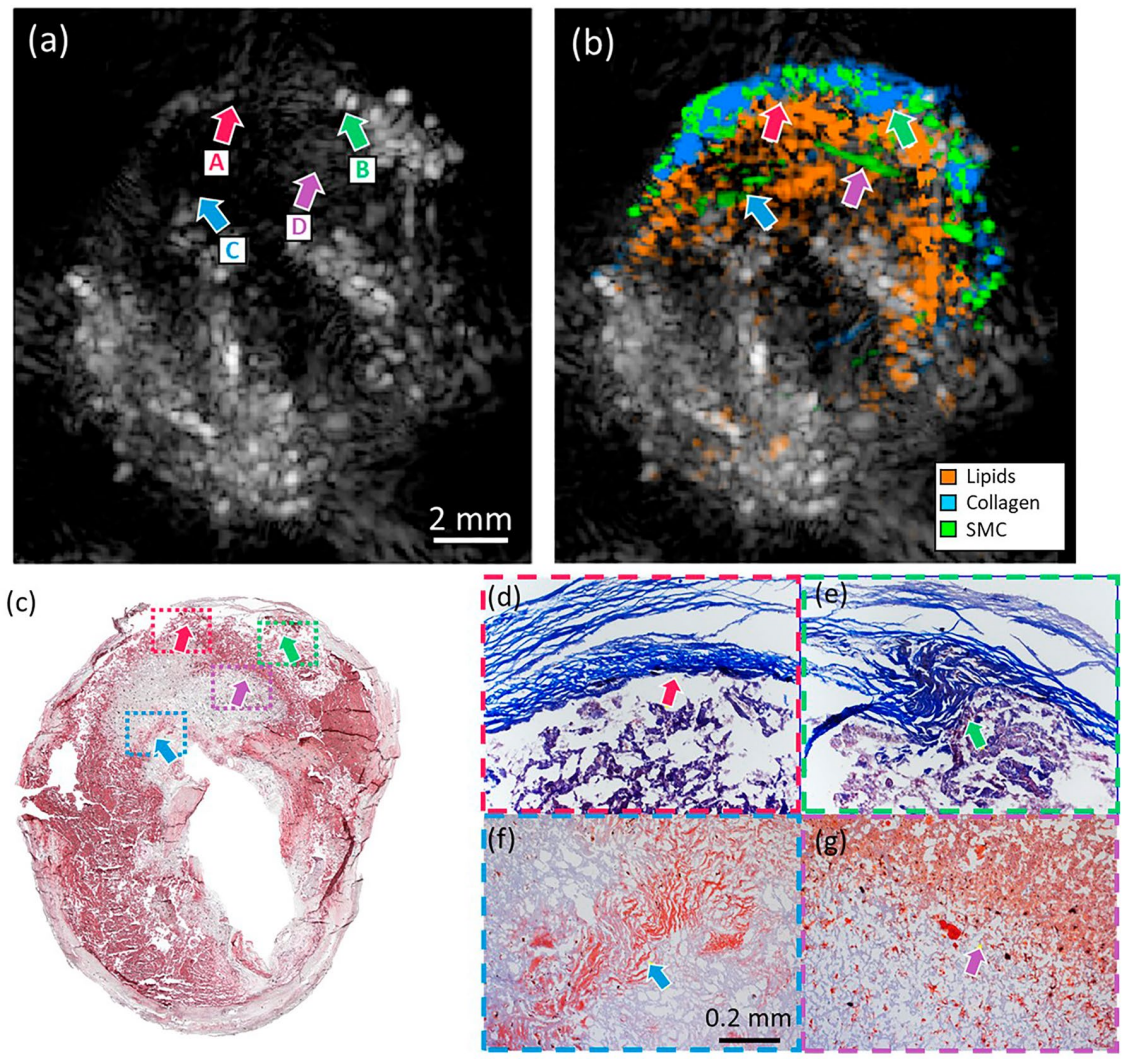
Endarterectomy plaque with a large necrotic area. (**a**,**b**) are the US and proportion maps for collagen, lipids, and SMC using selected wavelengths. (**c**) is the ORO staining of the plaque, where the red area indicates the presence of lipids. (**d**,**e**) are MT stains (magnification × 100), where the blue areas correspond to collagen; and arrows A and B point at geometrical features that are distinguishable in both histology and spectral unmixing map. (**f**,**g**) are ORO stains (magnification × 100), and arrows C and D point at the interfaces between lipid and lipid-free areas, observable in the unmixing proportions map.

Figure 5 presents another plaque characterized by a large lipid pool with necrotic areas, commonly regarded as prone to rupture. Arrows A-D indicates the location of several regions of interest, and their corresponding histology images are shown in Fig. 5d–g. Arrows A and B point at collagen-rich areas that are visible in the proportion map (Fig. 5b) and the MT histology (Fig. 5d,e). A collagen inclusion in the lipid pool can be observed in Fig. 5b,e, illustrating once again the detailed morphological assessment that can be achieved with our approach. In Fig. 5b, we can see an extensive speckle-like region identified as lipids that spreads through the sample and correlates with the ORO histology in Fig. 5c. Arrows C and D point at the interfaces between lipid-rich and lipid-free areas identified as SMC. Despite the low relative amplitude of lipids compared to other chromophores, it can be detected across the lesion apart from thin interfaces.

**Figure 6:**
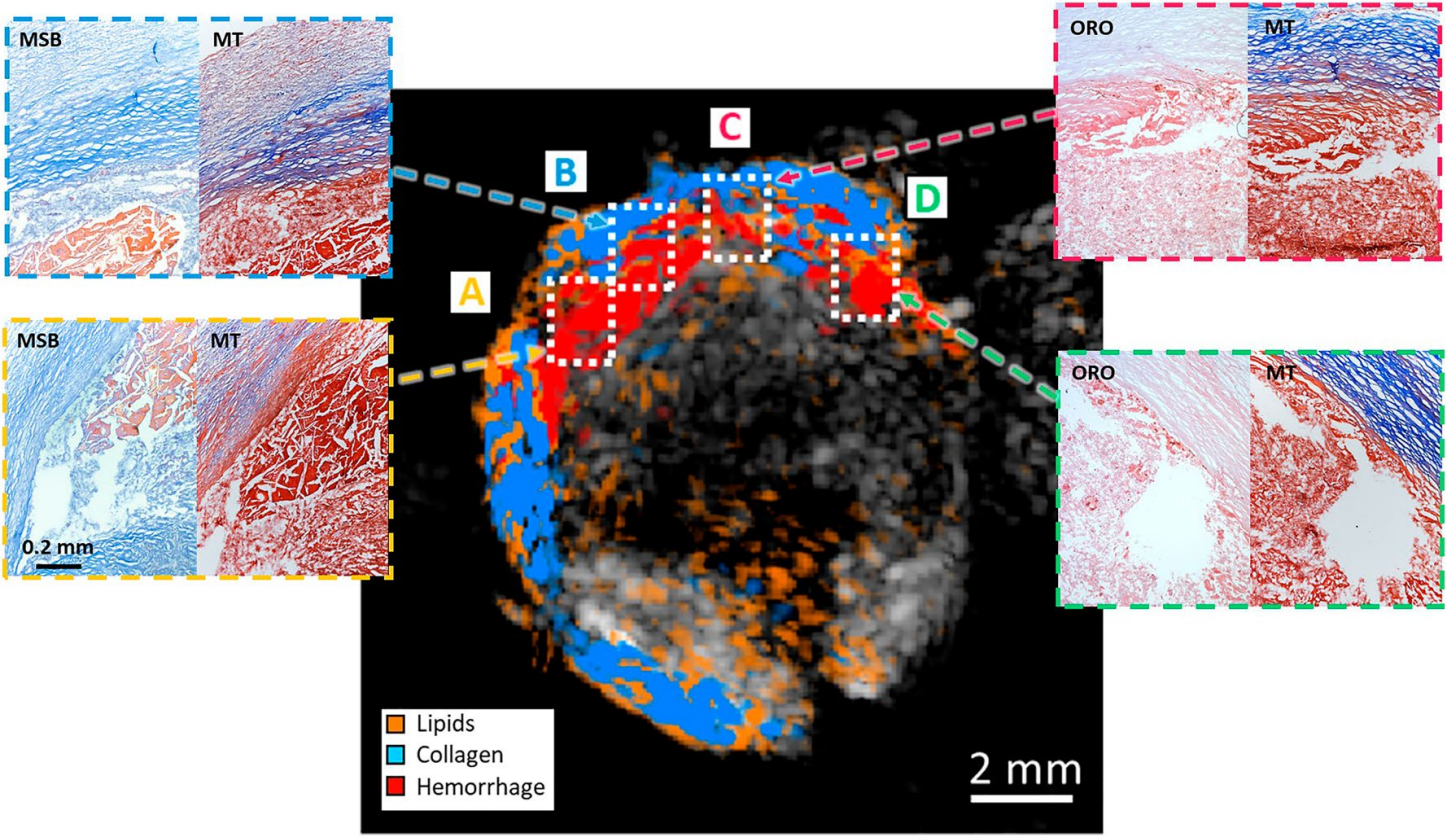
MSPAI unmixing of Intra-plaque hemorrhage sample sing selected wavelengths. White boxes in the proportions image indicate the location of the histology images. There is a good correlation between the collagen predictions with the MT staining. Hemorrhage (mainly methemoglobin) regions correlate with the fibrin regions (orange) in MSB for regions A and B, and with the empty areas in regions C and D, typically due to blood leaking out during sample preparation.

Figure 6 shows the proportion map obtained from a plaque with an intra-plaque hemorrhage, where the main detected constituents are collagen, lipids, and hemorrhage. The spectral features from the region classified as hemorrhage indicate that it is constituted mainly of methemoglobin. Martius Scarlet Blue (MSB) Histology images in regions A to D show the presence of fibrin and leaked blood areas that coincide with the hemorrhage location. Furthermore, MT and ORO histology images show the presence of a collagen layer in the outer part of the plaque, and lipids spread through the plaque core. The presented results clearly exemplify the capability of MSPAI to image and identify the composition of different plaques morphologies.

## Discussion

In this study, we presented a blind unmixing approach for MSPAI, based on endmember detection, that can identify multiple components in human plaques and may improve the assessment of vulnerability in plaques. After analyzing the endmembers detected from PA data acquired in the designed plaque phantom and nine human plaques, we found that four endmembers had a high correlation over different acquisitions. By comparing the mean representation of the spectral modulation observed in the unmixed endmembers and the reference spectra in the literature, we can interpret the detected endmembers to the tissue composition of collagen, lipid, hemorrhages, and SMC, which are highly relevant to the assessment of plaque vulnerability. In general, all the unmixed results agree with the corresponding histological staining.

Two of the classifications, hemorrhage and lipids are indicators of plaque vulnerability and potential markers to assess therapeutic efficacy and disease progression. The spectrum classified as hemorrhage mainly presents the spectral features of methemoglobin and generates high-intensity signals, making them easily recognized. Besides, hemorrhage regions display a strong diffuse signal in contrast with the images expected from luminal blood^17^. Similarly, lipids signals were mainly observed as speckle-like regions and usually overlapped with other materials. As expected, the typical absorption peak of lipids is found at around 1190 nm, while the spectral features of other chromophores were usually found in the visible range. It indicates that although lipids usually are mixed with other degraded plaque tissues, they can still be independently classified. The additional material was classified as smooth muscle cells based on the pathophysiology of plaques since it was present as a cellular matrix in most plaques^5, 11^. Lastly, the photoacoustic signature of collagen could be detected throughout the whole plaques. However, in those plaques with a large lipid pool, collagen would be detected predominantly in the outer part of the plaque. Recent studies indicate that collagen remodeling could play a fundamental role in the mechanical stability of plaques, suggesting that its identification could provide valuable information for vulnerability assessment^41–43^.

Unlike traditional linear unmixing methods, the method proposed does not require prior knowledge of the sample chromophores, and the number of endmembers can be easily determined by analyzing their normalized cross-correlation. Another major advantage of the method proposed is its capability to differentiate complex mixtures instead of pure signals, making it a valuable tool for studying highly heterogeneous samples, such as biological tissue. Moreover, the principal characteristic of our approach compared to other blind unmixing techniques in MSPAI is the solution of multiple convex models that are initialized using K-means clustering, generating an initial differentiation in the data based on intensity. Given the broad dynamic range and intrinsic bandwidth limitation of MSPAI, defining multiple models (starting with intensity) prevents low-contrast regions and photoacoustic speckles from being misclassified as background noise.

Additionally, we evaluated the method with a limited number of selected wavelengths, proving the stability and feasibility of such an approach for in-vivo applications where acquisition time, imaging wavelengths, and system complexity are usually restricted. Although there is a trade-off between the number of wavelengths used and the accuracy of material decomposition, we observed an overall error in the prediction on phantom below 10%. Note, however, that the prediction error in the phantom experiments is also due to poor sampling of the transition between filled materials due to its discrete distribution, which is not the case in human plaque tissue. Therefore, such error would be less significant in the human plaques, which was confirmed by the comparison between the unmixing results in human plaques and histology.

While promising results have been acquired, some challenges remain, especially aiming for in-vivo applications. Bearing in mind that surrounding tissue will be present for in-vivo applications, we will perform further studies with more complex surrounding tissue around the plaque to understand and compensate for the effect of muscle, fat, and skin on the spectral coloring of PA signals. Besides, strongly scattering media will decrease the signal amplitude reducing the SNR and the penetration depth. This problem requires the development of better illumination schemes and intensity compensation algorithms, fields that develop in parallel to unmixing research. Nevertheless, we believe the multiple-model approach has proved to have good performance even under the effect of spectral coloring and considerable variation in the signal intensity between components. Please note that the wavelengths used in this paper were selected for an in-vitro setting and may not be suitable for in vivo applications. To be able to apply the method in-vivo, further modifications of the proposed method, such as constraining the wavelengths, repeating the wavelength selection procedure in a relevant in-vivo setting, and the improvement of the PA acquisition system (such as optimizing the probe design) are likely required. Finally, we must mention that the scope of our unmixing approach on MSPAI is not limited to the materials presented in this study. Other components may be identified by improving the system resolution, spectral sampling, and SNR, which may lead to the identification of subtypes of collagen and lipids.

## Methods

### Phantom preparation

The phantom was prepared using a solution of 15wt% PVA (Sigma-Aldrich Mowiol 28-99, Burlington-Massachusetts, USA) and 85wt% water and three freezing-thaw cycles to achieve a consistent stiffness. The channels were filled with three biological materials: oleate cholesterol (Sigma-Aldrich C9253-100MG, Burlington-Massachusetts, USA); plaque tissue was obtained from the outer part of an endarterectomy plaque; porcine blood from a local butcher house prepared with a solution of 10 wt% sodium citrate tribasic dihydrate and 90 wt% blood.

### Endarterectomy plaques

We scanned nine endarterectomy plaques obtained at the Catharina Hospital in Eindhoven. The study was approved by the local Ethical Committee (MEC-U, Nieuwegein, The Netherlands) and conducted according to relevant guidelines and regulations. All patients gave their informed consent to participate in this study.

### Spectral unmixing

For the blind unmixing of the MSPAI data, we adapted a Piecewise Convex Multiple-Model Endmember method (PCOMMEND) by Zare et al.^34^. PCOMMEND is an iterative unmixing approach that employs the least square method to estimate the set of endmembers that best represent a hyperspectral image composed of convex domains. It solves the standard linear mixture model and imposes non-negative constraints for the endmembers and proportions to ensure physical meaning. PCOMMEND employs an initial clustering to define multiple convex regions, allowing the solution of complex hyperspectral sets. We adapted this property to reduce the estimation error due to bandwidth limitation and fluence artifacts that can lead to a non-convex solution space. After convergence, the endmembers obtained for the bandwidth-limited region are projected into the endmembers of the other model, and then the proportions are recalculated.

We use two clusters during initialization, one for the bandwidth-limited signal and another for the rest of the modulated signal. We use a regularization parameter a = 0.1 to constrain the size of each cluster. The number of endmembers of each sample was determined by considering six endmembers, the number of selected wavelengths, and then reducing the number while monitoring the normalized cross-correlation between endmembers to identify the number of independent components. Finally, the endmembers are classified based on their modulation by comparing them with the spectra of common plaque constituents, such as collagen^45^, different types of cholesterol^22^, methemoglobin for hemorrhages^46^, and previous acquisitions contrasted with histology for SMC.

### Wavelength selection

Our wavelength selection is based on the iterative approach presented by Luke et al.^39^; however, instead of using the smallest principal value, we employ the condition number as a criterion for the model as suggested by Mazhar et al.^38^.

We define the conditioning matrix by taking the endmembers from the blind unmixing for 162 wavelengths. Then, the condition number of the matrix is iteratively optimized to determine the set of wavelengths that gives a well-conditioned problem. We found that the optimal number of endmembers for the phantom data was six; therefore, the same number of wavelengths was selected. For the ex-vivo samples, a set of representative spectra acquired from the endarterectomy samples was used to create the observation matrix to be optimized. We identified four materials of interest, and the background accounts for five wavelengths. Through experimental observations, we found that a sixth wavelength significantly increases the performance of the blind unmixing by reducing uncertainty due to laser power variation. The addition of one more wavelength didn't yield any improvement in the plaque unmixing.

### Endarterectomy plaques imaging

We validate the method in nine endarterectomy plaques scanned under the same imaging conditions as the phantom. Although all samples were imaged for 162 wavelengths, we split the data into two sets to exemplify the method's performance with a limited number of wavelengths. Four plaques are used for the wavelength selection algorithm, and the other five are analyzed with the wavelengths selected. Because plaque composition is distinct from the materials used in the phantom, different selected wavelengths were obtained for both experiments. The selected wavelengths for plaque imaging are: [580 nm, 600 nm, 720 nm, 900 nm, 1100 nm, and 1200 nm].

### Data registration and validation

For the co-registration of the US-PA images and the histology images, we used a grid placed under the plaque to localize the imaging plane. Then, the same grid is employed to cut the sample into sections of 4 mm thickness with the imaging plane located in the center. Afterward, we compared the shape of the plaque in the US image with the hematoxylin and eosin (H&E) histology of sections.

Plaque measurements were compared with the histology sections. Three of the principal plaque constituents were validated using Masson's Trichrome (MT) for collagen (blue staining), Oil Red O (ORO) for lipids (red staining), and Martius Scarlet Blue (MSB) for blood and fibrin (orange-red staining). Even though neither technique provides quantitative information, it is possible to make a fair assessment of the similarity between the unmixing and the histology by investigating the correspondence of the identified materials and their approximate location.

## Conclusions

In conclusion, we have demonstrated the feasibility and advantages of using MSPAI to assess multiple components in carotid plaques with a limited number of wavelengths. The identified materials: hemorrhage, lipid, and collagen, have an immediate application in the assessment of plaque vulnerability and progression. To this end, we introduce a modified piecewise convex multiple-model endmember approach as an effective blind unmixing technique for highly heterogeneous biological samples, despite the presence of spectral coloring and bandwidth-limitation effects. Furthermore, we exemplify the possibility of wavelength selection based on the conditioning of the coefficient matrix of a linear model and its functionality on blind unmixing. In future studies, the performance of MSPAI will be studied in the presence of luminal blood and surrounding tissue as the next step towards in-vivo applications. Finally, we believe the methods presented in this work can be implemented in other spectroscopy modalities for biological samples, where the exact spectral absorption of the components is unknown, making it a universal approach for in vivo morphology assessment using MSPAI.

## Data Availability

Data underlying the results presented in this paper are not publicly available at this time but may be obtained from the authors upon reasonable request.

## References

[1] Gonzalez L, Trigatti BL (2017). Macrophage apoptosis and necrotic core development in atherosclerosis: A rapidly advancing field with clinical relevance to imaging and therapy. Can. J. Cardiol..

[2] van der Toorn JE (2022). Carotid plaque composition and prediction of incident atherosclerotic cardiovascular disease. Circ. Cardiovasc. Imaging.

[3] Sigala F, Oikonomou E, Alexis S, Galyfos G, Tousoulis D (2018). ScienceDirect Coronary versus carotid artery plaques. Similarities and differences regarding biomarkers morphology and prognosis. Curr. Opin. Pharmacol..

[4] Wasserman BA (2014). Morphology and composition in relation to incident cardiovascular events : The multi-ethnic study of atherosclerosis. Radiology.

[5] Poredos P, Gregoric ID, Jezovnik MK (2020). Inflammation of carotid plaques and risk of cerebrovascular events. Ann. Transl. Med..

[6] Bos D (2021). Atherosclerotic carotid plaque composition and incident stroke and coronary events. J. Am. Coll. Cardiol..

[7] Tomey MI, Narula J, Kovacic JC (2014). Advances in the understanding of plaque composition and treatment options: Year in review. J. Am. Coll. Cardiol..

[8] Maldonado N, Damien AK (2015). Imaging and analysis of microcalcifications and lipid/necrotic core calcification in fibrous cap atheroma. Int. J. Cardiovasc. Imaging.

[9] Fok PW (2012). Growth of necrotic cores in atherosclerotic plaque. Math. Med. Biol..

[10] Bentzon JF, Otsuka F, Virmani R, Falk E (2014). Mechanisms of plaque formation and rupture. Circ. Res..

[11] Hafiane A (2019). Vulnerable plaque, characteristics, detection, and potential therapies. J. Cardiovasc. Dev. Dis..

[12] Huang X (2010). Intraplaque hemorrhage is associated with higher structural stresses in human atherosclerotic plaques: An in vivo MRI-based 3d fluid-structure interaction study. Biomed. Eng. Online.

[13] Michel JB, Virmani R, Arbustini E, Pasterkamp G (2011). Intraplaque haemorrhages as the trigger of plaque vulnerability. Eur. Heart J..

[14] Jeney V, Balla G, Balla J (2014). Red blood cell, hemoglobin and heme in the progression of atherosclerosis. Front. Physiol..

[15] Virmani R (2005). Atherosclerotic plaque progression and vulnerability to rupture: Angiogenesis as a source of intraplaque hemorrhage. Arterioscler. Thromb. Vasc. Biol..

[16] Barnett H (1998). Benefit of carotid endarterectomy in patients with symptomatic moderate or severe stenosis. North American Symptomatic Carotid Endarterectomy Trial Collaborators. N. Engl. J. Med..

[17] Muller J-W (2021). Towards in vivo photoacoustic imaging of vulnerable plaques in the carotid artery. Biomed. Opt. Express.

[18] Golledge J (1999). Selection of patients for carotid endarterectomy. J. Vasc. Surg..

[19] Saam T (2005). Quantitative evaluation of carotid plaque composition by in vivo MRI. Arterioscler. Thromb. Vasc. Biol..

[20] Hetterich H (2016). AHA classification of coronary and carotid atherosclerotic plaques by grating-based phase-contrast computed tomography. Eur. Radiol..

[21] Obaid DR (2013). Atherosclerotic plaque composition and classification identified by coronary computed tomography: Assessment of computed tomography-generated plaque maps compared with virtual histology intravascular ultrasound and histology. Circ. Cardiovasc. Imaging.

[22] Kotsugi M (2020). Lipid core plaque distribution using near-infrared spectroscopy is consistent with pathological evaluation in carotid artery plaques. Neurol. Med. Chir..

[23] Li, C. *et al.* Student Research Award in the Undergraduate Degree Candidate Category, 28th Annual Meeting of the Society for Biomaterials, Tampa, FL, April 24–27, 2002 Biochemical characterization of atherosclerotic plaque constituents using FTIR spectroscopy and. *Contract* (2002).

[24] Dima A, Ntziachristos V (2012). Non-invasive carotid imaging using optoacoustic tomography. Opt. Express.

[25] Merčep E, Deán-Ben XL, Razansky D (2018). Imaging of blood flow and oxygen state with a multi-segment optoacoustic ultrasound array. Photoacoustics.

[26] Ivankovic I, Merčep E, Schmedt CG, Deán-Ben XL, Razansky D (2019). Real-time volumetric assessment of the human carotid artery: Handheld multispectral optoacoustic tomography. Radiology.

[27] Attia ABE (2019). A review of clinical photoacoustic imaging: Current and future trends. Photoacoustics.

[28] Taki, A., Kermani, A., Ranjbarnavazi, S. M. & Pourmodheji, A. *Overview of Different Medical Imaging Techniques for the Identification of Coronary Atherosclerotic Plaques**Computing and Visualization for Intravascular Imaging and Computer-Assisted Stenting* (Elsevier Inc, 2017).

[29] Kubo T, Xu C, Wang Z, Van Ditzhuijzen NS, Bezerra HG (2011). Plaque and thrombus evaluation by optical coherence tomography. Int. J. Cardiovasc. Imaging.

[30] Fadhel MN, Hysi E, Assi H, Kolios MC (2020). Fluence-matching technique using photoacoustic radiofrequency spectra for improving estimates of oxygen saturation. Photoacoustics.

[31] Tzoumaz S, Ntziachristos V (2017). Spectral unmixing techniques for optoacoustic imaging of tissue pathophysiology. Philos Trans. R. Soc. A.

[32] Maturi M, Armanetti P, Menichetti L, Franchini MC (2021). An application of multivariate data analysis to photoacoustic imaging for the spectral unmixing of gold nanorods in biological tissues. Nanomaterials.

[33] Wang P, Wang P, Wang H-W, Cheng J-X (2012). Mapping lipid and collagen by multispectral photoacoustic imaging of chemical bond vibration. J. Biomed. Opt..

[34] Zare A, Gader P, Bchir O, Frigui H (2013). Piecewise convex multiple-model endmember detection and spectral unmixing. IEEE Trans. Geosci. Remote Sens..

[35] Dana N, Sowers T, Karpiouk A, Vanderlaan D, Emelianov S (2017). Optimization of dual-wavelength intravascular photoacoustic imaging of atherosclerotic plaques using Monte Carlo optical modeling. J. Biomed. Opt..

[36] Nassif IA, Zhou X, Yücel YH, Toronov V (2018). Wavelength optimization in the multispectral photoacoustic tomography of the lymphatic drainage in mice. Photoacoustics.

[37] Corlu A (2003). Uniqueness and wavelength optimization in continuous-wave multispectral diffuse optical tomography. Opt. Lett..

[38] Mazhar A (2010). Wavelength optimization for rapid chromophore mapping using spatial frequency domain imaging. J. Biomed. Opt..

[39] Luke GP, Nam SY, Emelianov SY (2013). Optical wavelength selection for improved spectroscopic photoacoustic imaging. Photoacoustics.

[40] Tzoumas S (2016). Eigenspectra optoacoustic tomography achieves quantitative blood oxygenation imaging deep in tissues. Nat. Commun..

[41] Han R (2021). The correlation between collagen types and ultrasound feature score in evaluating the vulnerability of carotid artery plaque. Front. Cardiovasc. Med..

[42] Ghasemi M, Johnston RD, Lally C (2021). Development of a collagen fibre remodelling rupture risk metric for potentially vulnerable carotid artery atherosclerotic plaques. Front. Physiol..

[43] Wissing TB (2022). Tissue-engineered collagenous fibrous cap models to systematically elucidate atherosclerotic plaque rupture. Sci. Rep..

[44] Jacques SL (2013). Erratum: Optical properties of biological tissues: A review (Physics in Medicine and Biology (2013) 58). Phys. Med. Biol..

[45] Sekar SKV (2017). Diffuse optical characterization of collagen absorption from 500 to 1700 nm. J. Biomed. Opt..

[46] Meng F, Alayash AI (2017). Determination of extinction coefficients of human hemoglobin in various redox states. Anal. Biochem..

